# Getting Involved With Policy as a Graduate Student

**DOI:** 10.1093/geroni/igaa057.2381

**Published:** 2020-12-16

**Authors:** Haley Gallo

**Affiliations:** University of Southern California, Los Angeles, California, United States

## Abstract

The third speaker is University of Southern California PhD candidate Haley Gallo. Haley will discuss strategies for connecting research to policy as a graduate student, as well as aging-policy internship opportunities for graduate students, including the GSA’s Greg O’Neill Policy Internship. Haley’s research focuses on policies that promote the goals of the Age-Friendly Cities and Communities initiative. She is passionate about including older adults—particularly those from groups who are traditionally left out—in the development of research and policy that affects people of all ages.

